# Relationship between plasma leucine-rich α-2-glycoprotein 1 and urinary albumin excretion in patients with type 2 diabetes

**DOI:** 10.3389/fendo.2023.1232021

**Published:** 2023-10-17

**Authors:** Jin Ook Chung, Seon-Young Park, Dong Hyeok Cho, Dong Jin Chung, Min Young Chung

**Affiliations:** ^1^ Division of Endocrinology and Metabolism, Department of Internal Medicine, Chonnam National University Medical School, Gwangju, Republic of Korea; ^2^ Division of Gastroenterology and Hepatology, Department of Internal Medicine, Chonnam National University Medical School, Gwangju, Republic of Korea

**Keywords:** albuminuria, biomarkers, diabetes mellitus, type 2, diabetic nephropathies, LRG1 protein

## Abstract

**Aims:**

To explore the relationship between plasma leucine-rich α-2-glycoprotein 1 (LRG1) level and the degree of urinary albumin excretion in patients with type 2 diabetes.

**Methods:**

We evaluated 332 patients with type 2 diabetes in a cross-sectional study.

**Result:**

The plasma LRG1 level differed significantly according to the quartiles of urinary albumin excretion (Q1 [<7.7 mg/g], 17.1 μg/mL; Q2 [7.7–15.0 mg/g], 17.5 μg/mL; Q3 [15.1–61.4 mg/g], 18.6 μg/mL; Q4 [≥61.5 mg/g], 22.3 μg/mL; *p* for trend = 0.003) under adjustment with other covariates. A positive correlation was found between plasma LRG1 level and urinary albumin excretion (ρ = 0.256, *p <*0.001). According to a multivariate model, the association between LRG1 and urinary albumin excretion remained significant, under adjustment for confounding factors (β = 0.285, *p <*0.001).

**Conclusion:**

Plasma LRG1 level was independently associated with urinary albumin excretion in patients with type 2 diabetes. This study suggests that LRG1 may be associated with increased excretion of urinary albumin in the early stages of diabetic nephropathy.

## Introduction

Leucine-rich α-2-glycoprotein 1 (LRG1), a member of the leucine-rich repeat family, is a secreted glycoprotein (1) with multifunctional signaling properties (2). Its biological functions are still not fully clarified, although it may be implicated in angiogenesis and inflammation (3). Previous studies have shown that an increased concentration of plasma LRG1 was found in inflammatory diseases and cardiovascular diseases, suggesting it may be a potential biomarker (2, 4, 5). Additionally, LRG1 may also be associated with kidney injury (2). In patients with chronic kidney disease, plasma LRG1 level was suggested to be associated with chronic kidney disease progression (6). A longitudinal study of patients with type 2 diabetes mellitus (T2DM) reported that an increased level of plasma LRG1 was associated with albuminuria and chronic kidney disease progression. However, it has not been clarified whether LRG1 is implicated in early-stage diabetic nephropathy in T2DM patients.

Diabetic nephropathy is a leading cause of end-stage renal disease (7), with one of the predominant clinical manifestations being albuminuria, which reflects kidney damage (7, 8). Albuminuria is known to be a significant risk factor for end-stage renal disease and cardiovascular morbidity and mortality in patients with diabetes (9, 10). In addition, recent studies suggest that subtle increases in urinary albumin excretion below the conventional albuminuria threshold (30 mg/g) are associated with an increased risk of cardiovascular events and mortality (11, 12). Moreover, increased urinary albumin excretion within normoalbuminuria has been associated with an increased risk of end-stage renal disease in diabetic populations (13). Therefore, because LRG1 is linked to kidney injury, it is important to evaluate whether LRG1 influences urinary albumin excretion in the normoalbuminuric as well as in the albuminuric range.

This study aimed to investigate the relationships between LRG1 level and degree of urinary albumin excretion in patients with T2DM, while also evaluating the relationships with other clinical indices of patients with T2DM.

## Materials and methods

### Subjects

A total of 332 individuals with T2DM, enrolled consecutively from our hospital’s diabetes clinic, were evaluated in the cross-sectional study. T2DM was diagnosed in line with the expert committee’s report on the diagnosis and classification of diabetes mellitus (14). Subjects who used glucocorticoids or had recent infection, stroke, heart failure, coronary artery disease, peripheral artery disease, chronic liver disease, nondiabetic renal disease, end-stage renal disease, or malignant tumors were excluded. Subjects who had asthma, rheumatoid arthritis, acute appendicitis and other acute and chronic inflammatory disease or immune disease were also excluded. Hypertension was diagnosed if the patient had a blood pressure of 140/90 mmHg or higher or was taking antihypertensive agents, while hyperlipidemia was assumed if the patient had a total cholesterol level of ≥6.5 mmol/L, triglyceride level ≥2.3 mmol/L, or was using lipid-lowering agents. The study was approved by an ethics committee of Chonnam National University Hospital (No. CNUH-2019-228), and informed consent was acquired from all participants.*Measurements*.

Venous blood samples were collected after overnight fasting. Glycated hemoglobin levels (HbA_1c_) were determined using ion-exchange liquid chromatography (Tosoh, Tokyo, Japan), while creatinine levels were measured using the Jaffe method. Plasma LRG1 level was measured by a sandwich enzyme-linked immunosorbent assay kit (Immuno-Biological Laboratories, Hamburg, Germany) following the manufacturer’s instructions. The reported intra- and interassay coefficients of variation were 3.0%–4.9% and 4.2%–5.1%, respectively. Urinary albumin excretion was measured using the urinary albumin-to-creatinine ratio. The mean values for urinary albumin excretion were determined from two spot urine collections acquired on two consecutive mornings. Albuminuria was defined as urinary albumin excretion ≥30 mg/g, while normoalbuminuria was <30 mg/g. The glomerular filtration rate was estimated using the Chronic Kidney Disease Epidemiology Collaboration equation (15).

### Statistical analyses

Data are expressed as the mean ± standard deviation (SD), median (interquartile range), or frequency (percentage) unless otherwise indicated. The test for linear trend across increasing categories of plasma LRG1 level treated categories as continuous variables in the model. A linear-by-linear association test was conducted for qualitative parameters and Spearman’s rank correlation analysis was used to evaluate the association between plasma LRG1 level and other clinical variables. An analysis of covariance (ANCOVA) was conducted to compare the average LRG1 level according to the quartiles of urinary albumin excretion. Plasma LRG1 was analyzed after logarithmic transformation because of a skewed distribution. We evaluated the association between LRG1 level and urinary albumin excretion using a multiple linear regression model. Model 1 was adjusted for age and sex; Model 2 was adjusted for age, sex, smoking, body mass index, systolic blood pressure (BP), hyperlipidemia, high-sensitivity C-reactive protein (hs-CRP), and use of angiotensin II receptor blocker (ARB)/angiotensin-converting enzyme inhibitor (ACE*i*); Model 3 was adjusted for the variables in Model 2 plus the duration of diabetes, HbA_1c_, and use of OHAs and insulin. The variance inflation factor was used to evaluate multicollinearity. A variance inflation factor > 5 was excluded from the model. All statistical analyses were performed using SPSS version 23.0 (SPSS, Inc.). A *p*-value of <0.05 denoted statistical significance.

## Results

The clinical characteristics of the study participants are presented in **Table 1**. The systolic BP, DM duration, HbA_1c_ level, and prevalence of hyperlipidemia and ARB/ACE*i* use tended to increase in patients with higher urinary albumin excretion, while estimated glomerular filtration rate tended to be lower in patients with increased albumin excretion (**Table 1**). LRG1 level also generally increased from the lowest quartile to the highest quartile of urinary albumin excretion. In addition, age, triglyceride level, and the prevalence of hypertension and insulin use differed significantly according to the quartiles of urinary albumin excretion.

**Table 1:** Characteristics of patients with T2DM according to the quartiles of urinary albumin excretion.

	Urinary albumin excretion (mg/g)				*p* for trend
	Quartile 1 (<7.7)	Quartile 2 (7.7–15.0)	Quartile 3 (15.1–61.4)	Quartile 4 (≥61.5)	
n	82	84	83	83	
Urinary albumin excretion (mg/g)	5.4 (4.2, 6.5)	10.8 (8.9, 12.0)	27.9 (20.3, 41.8)	176.6 (99.0, 590.8)	<0.001
Age (years)	55.9 ± 13.1	60.8 ± 12.6	62.7 ± 12.6	59.7 ± 3.5	0.040
Smoking, n (%)	15 (18.3)	7 (8.3)	10 (12.0)	13 (15.7)	0.814
Men, n (%)	52 (63.4)	37 (44.0)	43 (51.8)	51 (61.4)	0.915
BMI (kg/m^2^)	26.0 ± 3.7	25.7 ± 5.4	26.6 ± 4.1	25.8 ± 5.1	0.834
Hyperlipidemia, n (%)	58 (70.7)	69 (82.1)	69 (83.1)	70 (84.3)	0.034
Hypertension, n (%)	43 (52.4)	55 (65.5)	52 (62.7)	61 (73.5)	0.011
Systolic BP (mmHg)	127.7 ± 16.5	133.0 ± 15.9	133.6 ± 16.8	140.6 ± 19.6	<0.001
Diastolic BP (mmHg)	75.1 ± 11.5	76.2 ± 11.0	76.5 ± 11.5	78.4 ± 12.8	0.081
DM duration (years)	1.3 (0.1, 4.8)	5.0 (0.8, 12.0)	10.0 (3.5, 19.0)	12.0 (4.0, 19.0)	<0.001
HbA_1c_ (%)	7.1 ± 1.4	7.3 ± 1.5	8.3 ± 2.1	8.6 ± 2.2	<0.001
HbA_1c_ (mmol/mol)	55 ± 15	57 ± 16	67 ± 23	71 ± 24	<0.001
Triglyceride (mmol/L)	1.3 (0.9, 1.9)	1.1 (0.8, 1.7)	1.4 (1.0, 2.0)	1.5 (1.0, 2.2)	0.026
TC (mmol/L)	4.5 ± 1.3	4.2 ± 1.0	4.1 ± 1.2	4.4 ± 1.5	0.460
LDL-C (mmol/L)	2.7 ± 0.9	2.4 ± 0.7	2.4 ± 0.8	2.5 ± 1.0	0.349
HDL-C (mmol/L)	1.3 ± 0.3	1.3 ± 0.4	1.3 ± 0.3	1.3 ± 0.3	0.350
LRG1 (µg/mL)	13.6 (11.2, 17.4)	14.7 (11.5, 18.4)	17.2 (13.0, 23.7)	19.7 (12.8, 28.1)	<0.001
Estimated GFR (ml/min/1.73m^2^)	96.4 ± 14.0	92.5 ± 18.6	86.9 ± 22.2	77.3 ± 28.8	<0.001
hs-CRP (mg/dL)	0.05 (0.02, 0.16)	0.04 (0.02, 0.12)	0.07 (0.03, 0.20)	0.07 (0.03, 0.19)	0.170
Injectable agents					
Insulin, n (%)	11 (13.4)	10 (11.9)	20 (24.1)	26 (31.3)	0.001
GLP1-RA, n (%)	0 (0.0)	1 (1.2)	0 (0.0)	0 (0.0)	NA
Oral agents					
OHAs, n (%)	49 (59.8)	59 (70.2)	53 (63.9)	50 (60.2)	0.827
Sulfonylurea, n (%)	16 (19.5)	25 (29.8)	29 (34.9)	24 (28.9)	0.135
Metformin, n (%)	44 (53.7)	54 (64.3)	49 (59.0)	42 (50.6)	0.547
DPP-4*i*, n (%)	27 (32.9)	34 (40.5)	35 (42.2)	34 (41.0)	0.286
TZD, n (%)	4 (4.9)	6 (7.1)	9 (10.8)	9 (10.8)	0.115
SGLT2*i*, n (%)	9 (11.0)	13 (15.5)	10 (12.0)	12 (14.5)	0.678
ARB/ACE*i*, n (%)	28 (34.1)	38 (45.2)	43 (51.8)	50 (60.2)	0.001
CCB, n (%)	15 (18.3)	17(20.2)	17 (20.5)	24 (28.9)	0.115
Diuretics, n (%)	3 (3.7)	6 (7.1)	7 (8.4)	8 (9.6)	0.133
Beta-blocker, n (%)	0 (0.0)	1 (1.2)	0 (0.0)	1 (1.2)	NA
Statin, n (%)	52 (63.4)	65 (77.4)	64 (77.1)	60 (72.3)	0.235
Fibrate, n (%)	5 (6.1)	3 (3.6)	2 (2.4)	2 (2.4)	0.185
Ezetimibe, n (%)	6 (7.3)	9 (10.7)	13 (15.7)	8 (9.6)	0.440

Data = mean ± standard deviation or median (interquartile range).

ARB/ACEi, angiotensin II receptor blocker/angiotensin-converting enzyme inhibitor; BMI, body mass index; BP, blood pressure; CCB, calcium channel blocker; DPP-4i, dipeptidyl peptidase 4 inhibitor; eGFR, estimated glomerular filtration rate; GLP1-RA, glucagon-like peptide-1 receptor agonist; HbA_1c_, glycated hemoglobin; HDL-C, high-density lipoprotein cholesterol; hs-CRP, high-sensitivity C-reactive protein; LRG1, leucine-rich α-2-glycoprotein 1; LDL-C, low-density lipoprotein cholesterol; OHA, oral hypoglycemic agent; T2DM, type 2 diabetes mellitus; SGLT2i, sodium-glucose cotransporter 2 inhibitor; TC, total cholesterol; TZD, thiazolidinedione; NA, not applicable.

Selected significant results of the Spearman correlation analysis for LRG1 and other clinical indices in T2DM patients are shown in **Table 2**. Plasma LRG1 level in all T2DM patients correlated positively with age (ρ = 0.144, *p* = 0.009), DM duration (ρ = 0.174, *p* = 0.001), HbA_1c_ level (ρ = 0.152, *p* = 0.006), and hs-CRP (ρ = 0.404, *p* < 0.001), and correlated negatively with total cholesterol (ρ = -0.123, *p* = 0.025), high-density lipoprotein cholesterol (ρ = -0.127, *p* = 0.020), low-density lipoprotein cholesterol (ρ = -0.112, *p* = 0.042), and estimated glomerular filtration rate (*ρ* = -0.175, *p* = 0.001). In the subgroups of diabetic patients categorized by albuminuria, a significant correlation of LRG1 with DM duration, total cholesterol, and hs-CRP was only found in subgroups of T2DM patients who did not have albuminuria. A significant correlation of LRG1 with estimated glomerular filtration rate and hs-CRP was only found in the subgroup of T2DM patients with albuminuria. For all T2DM patients, a significantly positive correlation was found between LRG1 and urinary albumin excretion (ρ = 0.256, *p* < 0.001). The same significant correlation was also found in the T2DM patient subgroups with normoalbuminuria and albuminuria.

**Table 2:** Significant correlations between LRG1 and clinical variables in patients with T2DM.

	All T2DM patients^†^ (n= 334)	*p*-value	T2DM with normoalbuminuria^†^ (n= 212)	*p-* value	T2DM with albuminuria^†^ (n=120)	*p-* value
Age	0.144	0.009	0.129	0.061	0.168	0.066
DM duration	0.174	0.001	0.179	0.009	0.102	0.120
HbA_1c_	0.152	0.006	0.104	0.133	0.132	0.152
TC	-0.123	0.025	-0.144	0.036	-0.077	0.404
HDL-C	-0.127	0.020	-0.121	0.080	-0.073	0.429
LDL-C	-0.112	0.042	-0.118	0.087	-0.086	0.352
Urinary albumin excretion	0.256	<0.001	0.225	0.001	0.214	0.019
Estimated GFR	-0.175	0.001	-0.043	0.529	-0.302	0.001
hs-CRP	0.404	<0.001	0.421	<0.001	0.342	<0.001

^†^The value indicates Spearman’s rank correlation coefficient.

GFR, glomerular filtration rate; HbA_1c_, glycated hemoglobin; HDL-C, high density lipoprotein cholesterol; hs-CRP, high-sensitivity C-reactive protein; LDL-C, low density lipoprotein cholesterol; LRG1, leucine-rich α-2-glycoprotein 1; T2DM, type 2 diabetes mellitus; TC, total cholesterol.

**Table 3** shows the average [95% confidence interval (CI)] plasma LRG1 level according to the degree of urinary albumin excretion. The average LRG1 level differed significantly according to the quartiles of urinary albumin excretion (Q1 [<7.7 mg/g], 17.1 μg/mL, 95% CI 15.0–19.1; Q2 [7.7–15.0 mg/g], 17.5 μg/mL, 95% CI 15.5–19.4; Q3 [15.1–61.4 mg/g], 18.6 μg/mL, 95% CI 16.6–20.6; Q4 [≥61.5 mg/g], 22.3 μg/mL, 95% CI 20.3–24.4; *p* for trend = 0.003), after being adjusted for age, sex, smoking, body mass index, systolic BP, hyperlipidemia, hs-CRP, duration of diabetes, HbA_1c_, use of ARB/ACE*i*, OHAs and insulin.

**Table 3:** Comparison of means of LRG1 depending on the degree of urinary albumin excretion in patients with T2DM.

	Urinary albumin excretion (mg/g)	Model 1	Model 2	Model 3
		Mean (95% CI)	Mean (95% CI)	Mean (95% CI)
LRG1^†^ (µg/mL)	Quartile 1 (<7.7)	16.5 (14.0–19.0)	16.4 (14.4–18.4)	17.1 (15.0–19.1)
	Quartile 2 (7.7–15.0)	16.2 (13.8–18.6)	17.0 (15.1–18.9)	17.5 (15.5–19.4)
	Quartile 3 (15.1–61.4)	18.9 (16.4–21.3)	18.9 (17.0–20.9)	18.6 (16.6–20.6)
	Quartile 4 (≥61.5)	23.8 (21.4–26.2)	23.0 (21.0–25.0)	22.3 (20.3–24.4)
	*P* for trend	*p <*0.001	*p <*0.001	*p* = 0.003

Data are mean (95% CI). ^†^Log-transformed before analysis.

Model 1: adjusted for age and sex.

Model 2: additionally adjusted for smoking, body mass index, systolic BP, hyperlipidemia, hs-CRP^†^, and use of ARB/ACEi.

Model 3: adjusted for variables in model 2 and diabetes duration^†^, HbA_1c_, use of OHAs and insulin.

ARB/ACEi, angiotensin II receptor blocker/angiotensin-converting enzyme inhibitor; BP, blood pressure; CI, confidence interval; HbA_1c_, glycated hemoglobin; hs-CRP, high-sensitivity C-reactive protein; OHAs, oral hypoglycemic agents; T2DM, type 2 diabetes mellitus.

We carried out multiple linear regression analysis to investigate independent associations (**Table 4**), which demonstrated that LRG1 level was an independent determinant for urinary albumin excretion, after being adjusted for age, sex, smoking, body mass index, systolic BP, hyperlipidemia, hs-CRP, duration of diabetes, HbA_1c_, use of ARB/ACE*i*, OHAs and insulin (β = 0.285, *p* < 0.001; **Table 4**). Alternatively, when hypertension was included as an independent variable in the models and systolic BP and ARB/ACE*i* use were excluded, LRG1 level was still associated with urinary albumin excretion (**Supplementary Table 1**).

**Table 4:** Multiple linear regression analysis for urinary albumin excretion^†^ in patients with T2DM.

	Unadjusted		Model 1		Model 2		Model 3	
	β	*p*-value	β	*p*-value	β	*p*-value	β	*p*-value
LRG1^†^	0.287	<0.001	0.309	<0.001	0.369	<0.001	0.285	<0.001
Age			-0.018	0.737	-0.091	0.105	-0.126	0.026
Sex			0.074	0.232	0.057	0.356	0.081	0.168
Smoking					0.032	0.551	-0.010	0.848
BMI					-0.042	0.446	0.006	0.916
Systolic BP					0.229	<0.001	0.223	<0.001
Hyperlipidemia					0.064	0.218	0.031	0.544
hs-CRP^†^					-0.128	0.031	-0.118	0.039
ARB/ACE*i*					0.131	0.017	0.100	0.060
HbA_1c_							0.177	0.001
DM duration^†^							0.221	0.001
Use of insulin							0.044	0.575
Use of OHAs							-0.060	0.443
R^2^ (adjusted R^2^)	0.083 (0.080)		0.093 (0.085)		0.193 (0.171)		0.289 (0.260)	

^†^Log-transformed before analysis. β, standardized partial regression coefficient.

Model 1: adjusted for age and sex.

Model 2: additionally adjusted for smoking, body mass index, systolic BP, hyperlipidemia, hs-CRP^†^, and use of ARB/ACEi.

Model 3: adjusted for variables in model 2 and diabetes duration^†^, HbA_1c_, use of OHAs and insulin.

ARB/ACEi, angiotensin II receptor blocker/angiotensin-converting enzyme inhibitor; BP, blood pressure; CI, confidence interval; HbA_1c_, glycated hemoglobin; hs-CRP, high-sensitivity C-reactive protein; OHAs, oral hypoglycemic agents; T2DM, type 2 diabetes mellitus.

## Discussion

In this study, we determined that plasma LRG1 level correlated positively with the degree of urinary albumin excretion and correlated negatively with the estimated glomerular filtration rate. In the individual groups, plasma LRG1 level was positively correlated with urinary albumin excretion in patients with albuminuria and normoalbuminuria. In addition, the multiple regression analysis identified that plasma LRG1 level was independently associated with urinary albumin excretion after adjustments for the conventional confounders.

LRG1 is a secretory glycoprotein with multifunctional properties in a context-dependent manner (2, 3). LRG1 is associated with pathological neovascularization (3). LRG1 is also thought to be involved in cell differentiation, adhesion, migration, and apoptosis (2, 3). Previous studies have noted a close association between LRG1 and cardiovascular disease, demonstrating that increased levels of circulating LRG1 were associated with increased risks for coronary artery disease, heart failure, and mortality (4, 5, 16, 17). These results suggest that LRG1 might be a potential biomarker for cardiovascular disease.

Recent studies have also suggested close associations between LRG1 and renal injury (2). Several studies have shown that LRG1 is expressed in glomerular endothelial cells and an increase in LRG1 expression is found in mouse and human diabetic kidneys (2, 18, 19). In a study of mouse models, genetic ablation of LRG1 attenuated diabetic glomerulopathy (19). In patients with chronic kidney disease, plasma LRG1 level was suggested to be associated with the progression of chronic kidney disease (6). In addition, several studies have reported that LRG1 may be implicated in diabetic nephropathy progression (2, 19, 20). For example, Hong et al. (19) reported that an increased LRG1 level was associated with adverse renal outcomes including a 40% decline in estimated glomerular filtration rate and end-stage renal disease in patients with T2DM, while Liu et al. (20) showed that plasma LRG1 level was associated with albuminuria progression in patients with T2DM. Moreover, we found that plasma LRG1 level was positively correlated with the degree of urinary albumin excretion in patients with T2DM, and in individual groups; this association was observed not only in the patients with albuminuria but also in those with normoalbuminuria. Our findings suggest, therefore, that LRG1 might be associated with increased excretion of urinary albumin in the early stages of diabetic nephropathy. However, further prospective investigations are necessary to validate our findings.

In the present study, we found that LRG1 was associated with glycemic status reflected by HbA_1c_ level and diabetes duration, which are important contributors to diabetic kidney injury (21). An *in vitro* study showed that LRG1 expression increased in response to hyperglycemic conditions (19). Previous studies have also suggested that increased LRG1 level is observed in patients with a longer duration of T2DM (5, 17). In addition, several studies have shown that LRG1 level is related to age and dyslipidemia (5, 17, 22, 23). Thus, the association between LRG1 level and urinary albumin excretion observed in our study might be partially accounted for by these factors, because these contribute to an increased risk of diabetic nephropathy as well (21). However, the relationship between LRG1 level and urinary albumin excretion remained statistically significant, under adjustment with these confounders in multivariable analyses, implying that these factors did not exert a significant impact on the association between LRG1 level and urinary albumin excretion.

Potential mechanisms could link LRG1 to diabetic nephropathy. First, increased angiogenesis is a trait of diabetic glomerulopathy (24) and occurs in the early stages of diabetic nephropathy (25). LRG1 has been suggested to be mitogenic to endothelial cells and to promote angiogenesis through modulating transforming growth factor (TGF)-β signaling (3). Enhanced abnormal angiogenesis is associated with the growth of immature new vessels (24), and these changes might be implicated in functional and structural abnormalities in glomerular endothelial cells, resulting in impaired filtration barrier and albumin leakage (24). Second, TGF-β accessory receptor endoglin, which LRG1 binds to, is also known to regulate endothelial nitric oxide synthase stability (26). Interaction between LRG1 and endoglin might disrupt the homeostasis of nitric oxide and disturb endothelial cell function (27). In a diabetic mouse model, endothelial nitric oxide synthase deficiency was found to be associated with accelerated nephropathy (28). Third, TGF-β is suggested to be a key regulator of fibrosis (29). Increased LRG1 levels may intensify TGF-β signaling, induce pro-fibrotic pathways, and result in progressive kidney injury (30). Finally, LRG1 is associated with inflammation (2), which is reported to have a critical role in progressive loss of kidney function (31).

CRP has been considered a marker of systemic inflammation (32). Although there is a large body of evidence suggesting connections between inflammation and diabetic nephropathy (31), the findings regarding relationships between CRP and diabetic nephropathy are inconsistent throughout the literature (33, 34). In this study, however, we found that the relationship between LRG1 level and urinary albumin excretion was statistically significant after adjusting for the confounders, including hs-CRP level, in multivariable analysis. As a consequence, our data suggest that LRG1 might be implicated in renal injury through mechanisms independent of CRP. However, because other factors as well as inflammatory stimuli can have an impact on CRP levels (32), further research in this regard is required.

This study has some limitations. First, because this study was performed in a single center and the participants were limited to one ethnic group, our results might not be generalized to other population with diabetes throughout the world. In addition, our results might not be applicable to other types of nephropathy. Second, information about the duration of drug use was not obtained in this study. Third, the causality of the associations between plasma LRG1 level and urinary albumin excretion could not be determined, because this was a cross-sectional study. Finally, even if several potential confounding variables were considered in the regression model, the relationship between LRG1 and urinary albumin excretion could be influenced by confounders that have not yet been measured (35). Despite these limitations, we believe that our findings might provide important information on the relationship between plasma LRG1 level and urinary albumin excretion in T2DM patients.

In conclusion, this study showed that plasma LRG1 level was independently associated with urinary albumin excretion in T2DM patients. Our results suggest that LRG1 level may be an early indicator of diabetic nephropathy in patients with T2DM. However, further large-scale longitudinal studies are necessary to elucidate the precise role of LRG1 in early-stage diabetic nephropathy.

## Data availability statement

The original contributions presented in the study are included in the article/**Supplementary Material**. Further inquiries can be directed to the corresponding author.

## Ethics statement

The studies involving humans were approved by an ethics committee of Chonnam National University Hospital. The studies were conducted in accordance with the local legislation and institutional requirements. The participants provided their written informed consent to participate in this study.

## Author contributions

JC designed the study, analyzed data, drafted the manuscript, and approved its final version. S-YP and MC contributed to the statistical analyses and interpretation of data. JC, D-HC, DC acquired data. All authors reviewed and approved the final manuscript.
