# Supplementary material for: Relationship between plasma leucine-rich α-2-glycoprotein 1 and urinary albumin excretion in patients with type 2 diabetes
Source: Front Endocrinol (Lausanne). 2023 Oct 17;14:1232021. doi: 10.3389/fendo.2023.1232021 (PMC10617030; doi:10.3389/fendo.2023.1232021)
Supplement: Supplementary file 1 [file Table_1.docx]

Supplementary Table 1. Multiple linear regression analysis for urinary albumin excretion^†^ in patients with T2DM when hypertension was included as an independent variable and systolic BP and use of ARB/ACE*i* were excluded

|  | Unadjusted | | Model 1 | | Model 2 | | Model 3 | |
| --- | --- | --- | --- | --- | --- | --- | --- | --- |
|  | β | *p*-value | β | *p*-value | β | *p*-value | β | *p*-value |
| LRG1^†^ | 0.287 | <0.001 | 0.309 | <0.001 | 0.374 | <0.001 | 0.288 | <0.001 |
| Age |  |  | -0.018 | 0.737 | -0.070 | 0.242 | -0.121 | 0.042 |
| Sex |  |  | 0.074 | 0.232 | 0.062 | 0.327 | 0.087 | 0.147 |
| Smoking |  |  |  |  | 0.016 | 0.771 | -0.024 | 0.656 |
| BMI |  |  |  |  | -0.008 | 0.891 | 0.039 | 0.701 |
| Hypertension |  |  |  |  | 0.121 | 0.037 | 0.106 | 0.057 |
| Hyperlipidemia |  |  |  |  | 0.072 | 0.184 | 0.026 | 0.618 |
| hs-CRP^†^ |  |  |  |  | -0.127 | 0.039 | -0.108 | 0.067 |
| HbA_1c_ |  |  |  |  |  |  | 0.152 | 0.005 |
| DM duration^†^ |  |  |  |  |  |  | 0.264 | <0.001 |
| Use of insulin |  |  |  |  |  |  | 0.052 | 0.526 |
| Use of OHAs |  |  |  |  |  |  | -0.052 | 0.518 |
| R^2^ (adjusted R^2^) | 0.083 (0.080) | | 0.093 (0.085) | | 0.129 (0.108) | | 0.237 (0.208) | |

^†^Log-transformed before analysis. β, standardized partial regression coefficient

Model 1: adjusted for age and sex

Model 2: additionally adjusted for smoking, body mass index, hypertension, hyperlipidemia, and hs-CRP^†^

Model 3: adjusted for variables in model 2 plus diabetes duration^†^, HbA_1c_, use of OHAs and insulin

HbA_1c_, glycated hemoglobin; hs-CRP, high-sensitivity C-reactive protein; OHAs, oral hypoglycemic agents; T2DM, type 2 diabetes mellitus
